# Murine Endogenous Retroviruses Are Detectable in Patient-Derived Xenografts but Not in Patient-Individual Cell Lines of Human Colorectal Cancer

**DOI:** 10.3389/fmicb.2018.00789

**Published:** 2018-04-24

**Authors:** Stephanie Bock, Christina S. Mullins, Ernst Klar, Philippe Pérot, Claudia Maletzki, Michael Linnebacher

**Affiliations:** ^1^Department of General Surgery, Molecular Oncology and Immunotherapy, University Medicine Rostock, Rostock, Germany; ^2^INSERM U1117, Biology of Infection Unit, Laboratory of Pathogen Discovery, Institut Pasteur, Paris, France

**Keywords:** mERV, expression, PDX, PDX-derived cell lines, CDX, colorectal cancer

## Abstract

Endogenous retroviruses are remnants of retroviral infections. In contrast to their human counterparts, murine endogenous retroviruses (mERV) still can synthesize infectious particles and retrotranspose. Xenotransplanted human cells have occasionally been described to be mERV infected. With genetic engineered mice and patient-derived xenografts (PDXs) on the rise as eminent research tools, we here systematically investigated, if different tumor models harbor mERV infections. Relevant mERV candidates were first preselected by next generation sequencing (NGS) analysis of spontaneous lymphomas triggered by colorectal cancer (CRC) PDX tissue. Two primer systems were designed for each of these candidates (AblMLV, EcoMLV, EndoPP, MLV, and preXMRV) and implemented in an quantitative real-time (RT-qPCR) screen using murine tissues (*n* = 11), PDX-tissues (*n* = 22), PDX-derived cell lines (*n* = 13), and patient-derived tumor cell lines (*n* = 14). The expression levels of mERV varied largely both in the PDX samples and in the mouse tissues. No mERV signal was, however, obtained from cDNA or genomic DNA of CRC cell lines. Expression of EcoMLV was higher in PDX than in murine tissues; for EndoPP it was the opposite. These two were thus further investigated in 40 additional PDX. In addition, four patient-derived cell lines free of any mERV expression were subcutaneously injected into immunodeficient mice. Outgrowing cell-derived xenografts barely expressed EndoPP. In contrast, the expression of EcoMLV was even higher than in surrounding mouse tissues. This expression gradually vanished within few passages of re-cultivated cells. In summary, these results strongly imply that: (i) PDX and murine tissues in general are likely to be contaminated by mERV, (ii) mERV are expressed transiently and at low level in fresh PDX-derived cell cultures, and (iii) mERV integration into the genome of human cells is unlikely or at least a very rare event. Thus, mERVs are stowaways present in murine cells, in PDX tissues and early thereof-derived cell cultures. We conclude that further analysis is needed concerning their impact on results obtained from studies performed with PDX but also with murine tumor models.

## Introduction

Retroviruses are reverse-transcriptase encoding viruses with a single-stranded RNA-genome. Endogenous retroviruses are located in somatic as well as germ cells and are therefore passed on to following generations. In contrast to their human equivalents, murine endogenous retroviruses (mERV) still have the ability to synthesize infectious particles and to retrotranspose. MERV are categorized according to their ability to infect foreign species, which is called host tropism, into ecotropic (not able to build infectious particle in original host, but replication is possible in thereof established cultures), xenotropic (infect only foreign species) and polytropic (infect original and as well foreign species) viruses (Stoye and Coffin, 1987; Weiss, 2006).

Xenotransplants of human tumor tissue grown in immunodeficient mice and termed patient-derived xenografts (PDXs) are used to expand the original tumor tissue and to exactly mimic the biological environment: the latter is hardly possible in cell culture. With the revival of PDX-models for research and especially for pharmaceutical drug development (Izumchenko et al., 2016), the question in dispute whether or not mERV are present and active in these preclinical tumor models demands a definite answer.

Several groups described mERV infections of human tumor tissues xenografted into immunodeficient mice and of thereof derived cell lines.

In 53% of independently isolated BALB/c nude mouse-derived PDX tissues of human breast cancer, a replication competent xenotropic endogenous murine leukemia virus (XMLV) was traceable (Naseer et al., 2015). Subsequent to xenotransplantation into SCID mice, an infection with B10 xenotropic virus 1 (Bxv1) was detectable in two human pancreatic cancer cell lines (Kirkegaard et al., 2016).

Zhang et al. (2011) described a 23% XMLV infection rate of PDX-derived cell lines by using TaqMan qPCR systems directed against XMLV gag, env, and pol regions. Surprisingly, they tested also 17% of primary cell lines XMLV positive; but failed to detect XMLV in cell lines generated in a lab not working with PDX-models (Zhang et al., 2011).

Furthermore, mERV may have oncogenic potential. Abelson murine leukemia virus (AblMLV) can induce pre-B-cell lymphoma (Yi and Rosenberg, 2008) and acute transforming retrovirus (AKT8) thymic lymphoma (Staal and Hartley, 1988). This would have significant consequences for general PDX usage and the results of PDX-based basic research as well as therapeutic drug testing and preclinical development. Thus, we took advantage of our large collection of patient-derived tumor models mainly established from colorectal cancer (CRC) to perform a comprehensive analysis of possible mERV infection of PDX, PDX-derived cell lines and primary tumor-derived cell lines.

In previous work, we occasionally observed the occurrence of lymphomas in NOD-SCID mice carrying human CRC PDX (Mullins et al., 2017). In two cases, lymphoma formation could be reproduced by implantation of a second piece of the primary patient tumor. Molecular and pathomorphological analysis revealed that the lymphoma cells were of murine origin (unpublished data). We hypothesized that it is likely that (human) tumor cell-derived factors trigger this lymphoma development. In light of the previously mentioned data on infections with mERV, we decided to additionally screen for the presence of potentially oncogenic murine viruses in these two CRC PDX tissues associated with lymphoma development in NOD-SCID mice.

## Materials and Methods

### Next Generation Sequencing (NGS)

RNA from the two PDX HROC32 and HROC39 were sequenced on an Illumina HiSeq2000 instrument in paired-end 2 × 100 nt. Raw read numbers were 80.3 × 10^6^ and 183.2 × 10^6^ for HROC32 and HROC39, respectively. After an initial step consisting of QC and trimming, reads were mapped to the human reference genome hg19 using Bowtie2. Unmapped reads were assembled with CLC Assembler into contigs of 100 nt minimal size. A search for viral and bacterial sequences was done from the contigs using a blastn procedure (best score) on NCBI databases genbank_release_virus and genbank_release_bacteria. From that, hits with an alignment longer than 60 nt were kept and their corresponding sequences were retrieved and used for a second (validation) blastn search, against the entire genbank database. Sequences for which the results were the same after the two blast searches were considered correctly assigned to a taxonomy.

### Xenotransplantation Procedures

Patient-derived xenografts tissues used in this study have been generated from CRC and glioblastoma patient tumors as described in previous studies using NMRI-*Foxn1*^nu^ (NMRI nu/nu), NOD.CB17-*Prkdc*^scid^/NcrCrl (NOD SCID) and NOD.Cg-*Prkdc*^scid^ *Il2rg*^tm1Wjl^/SzJ (NSG) mice (Linnebacher et al., 2010; William et al., 2017). To generate cell-derived xenotransplants, cells of patient-derived cell lines were suspended in 50 μl PBS and subcutaneously injected using a 1 ml syringe. A total amount of 1 × 10^6^ cells was injected dorsally, 3 × 10^6^ cells into the left and 5 × 10^6^ cells into the right flank. Outgrowing cell-derived xenograft (CDX) were explanted when at least one of the three tumors reached a size of 6–9 mm. Expression of EcoMLV and EndoPP in these tissues was subsequently analyzed using the probe systems.

### Cell Culture

Patient-derived cell lines of CRC patients (HROC24, HROC32, HROC39, HROC173, and HROC183) were cultured in DMEM/Ham’s F12 (1:1) supplemented with 10% fetal calf serum (FCS) and 2 mmol/l L-glutamine, as described before (Maletzki et al., 2012, 2015). Cell cultures established from the CDX were cultured initially in a coated 6-well plate and transferred at passage 1 into a T25 cell culture flask. A snap frozen cell pellet was prepared from this and subsequent passages for isolation of nucleic acids.

### RNA Isolation and cDNA Synthesis

RNA was isolated from cell pellets using EURx GeneMATRIX Cell Culture RNA Purification Kit (EURx; Gdańsk, Poland). Tissue samples were isolated using the Precellys Tissue RNA Kit (Peqlab; Erlangen, Germany). Skin samples were processed using TRIsure^TM^ according to the manufacturers’ description (Bioline GmbH; Luckenwalde, Germany). CDNA synthesis was performed using Reverase^®^ (Bioron, Ludwigshafen, Germany). Contamination of the isolated RNA by gDNA was minimized by incorporation of a DNAse step in all RNA isolation protocols used.

### Quantitative Real-Time PCR

Duplicate determination of mERV expression (AblMLV, EcoMLV, EndoPP, MLV, and preXMRV) was done by quantitative real-time PCR (qRT-PCR) on a ViiA^TM^ 7 Real-Time PCR System instrument (Thermo Fisher Scientific, Waltham, MA, United States). For the initial screening, two primer systems were designed for each mERV (**Table 1**). CDNA (stored at -30°C) was analyzed in duplicates in 12.5 μl reactions containing 10 μM of each primer, SYBR Green and reagents provided in the Fast SG qPCR Master Mix (Roboklon, Berlin, Germany) according to the manufacturer’s protocol. Cycling parameters were as follows: 2 min at 50°C, 10 min at 95°C and 40 cycles of 15 s at 95°C, 30 s at 60°C and afterward a melting curve analysis was performed. A non-template-control (NTC) was included in each qRT-PCR run and as housekeeping-genes murine GAPDH and human β-actin (**Table 2**) were used. For further investigations of Eco-MLV and Endo-PP, probe systems were designed (**Table 3**). The reagents were provided in a Fast Probe qPCR Master Mix (Roboklon) and applied according to the manufacturer’s protocol. Cycling parameters were as follows: 2 min at 50°C, 10 min at 95°C and 40 cycles of 15 s at 95°C and 30 s at 60°C.

**Table 1:** Polymerase chain reaction (PCR) primers, used for SYBR Green based qRT-PCR analysis.

Abelson (P160) murine leukemia virus (Ab-MLV) abl gene (X02963)	
**AblMLV-1**	
Forward primer	5′-TGT ACG AGG GCG TTT GGA AG-3′
Reverse primer	5′-GGG TAC ACA CCC CTA GCA G-3′
Product size	153 bp
Gene region	424–577 (gag-abl gene product, altered reading frame; includes only the abl part)
**AblMLV-2**	
Forward primer	5′-CTG GGA GAA AGC GAT GCA CT-3′
Reverse primer	5′-CGC TCA TCT TCA TTT AGG CTG C-3′
Product size	101 bp
Gene region	1323–1424 (gag-abl gene product, altered reading frame; includes only the abl part)

**Ecotropic murine leukemia virus (KJ668270)**	

**EcoMLV**	
Forward primer	5′-GTA ACG CCA TTT TGC AAG GC-3′
Reverse primer	5′-ATG AGG CTT GGG GTT GAT CC-3′
Product size	253 bp
Gene region	7864–8117 (long terminal repeat)

***Mus musculus* mobilized endogenous polytropic provirus (FJ544578)**	

**EndoPP-1**	
Forward primer	5′-GGC CGG AAC ACG AGT AAG AG-3′
Reverse primer	5′-TTG GCC ACC GAC TGA CTT AC-3′
Product size	273 bp
Gene region	4883–5156
**EndoPP-2**	
Forward primer	5′-TCT ATA GTC CCT GAG ACT GCC C-3′
Reverse primer	5′-CAA CCA GCA CTC TTG GGT TTT GT-3′
Product size	129 bp
Gene region	6527–6656 (env gene)

**Murine leukemia virus (AY714523)**	

**MLV**	
Forward primer	5′-GTA CCA ACA GGG TGT GGA GG-3′
Reverse primer	5′-TTG ATG TCA CTG GAG ACC GC-3′
Product size	179 bp
Gene region	587–766 (env gene)

**PreXMRV-2 (FR871850)**	

**preXMRV-1**	
Forward primer	5′-CTT AGA GAC CTC CGC TCC GT-3′
Reverse primer	5′-GGT TTG CCC TGA AAG ACC CA-3′
Product size	126 bp
Gene region	4584–4710 (gag-pro-pol gene)
**preXMRV-2**	
Forward primer	5′-TCC GTA CAC CTC CAG CTT TT-3′
Reverse primer	5′-TCT GTC TAG AAG CGC CCT CA-3′
Product size	205 bp
Gene region	4599–4804 (gag-pro-pol gene)


*For each mERV, two primer systems were designed, marked with “-1” and “-2”; albeit for EcoMLV and MLV only the primer system providing specific products is listed.*

**Table 2:** Polymerase chain reaction primers of housekeeping genes used in SYBR Green based qRT-PCR analysis.

Housekeeping genes	
Murine GAPDH	
Forward primer	5′-CAT GGC CTT CCG TGT TCC TA-3′
Reverse primer	5′-CCT GCT TCA CCA CCT TCT TGA-3′
Product size	104 bp
Human β-actin	
Forward primer	5′-CAT CGA GCA CGG CAT CGT CA-3′
Reverse primer	5′-TAG CAC AGC CTG GAT AGC AAC-3′
Product size	652 bp


**Table 3:** Probe systems used for EcoMLV and EndoPP detection.

Probe system EcoMLV	
Forward primer	5′-ACT TCT GCC CCA GCT AAC TG-3′
Reverse primer	5′-TGA TGG GTT TTA GGG ACC G-3′
Probe	5′-6-FAM-CCG GTC ACT TCG GAC AAG GTC A-BHQ-1-3′
Size	104 bp
Gene region	6862–6966 (env gene)
Probe system EndoPP	
Forward primer	5′–GCT CTC AAC CTC ACC AGT CC-3′
Reverse primer	5′-GGC AAC CCC TTC GTA GTA GG-3′
Probe	5′-6-FAM-CCG CTA CCA GAC ACA ACC AGC ACT-BHQ-1-3′
Size	84 bp
Gene region	6612–6695 (env gene)


Relative expression of EcoMLV and EndoPP in PDX tissues was calculated by using the following formula $\frac{2^{-\left(\text{Ct value relevent gene-Ct value murine GAPDH}\right)}}{\left(1+2^{-\left(\text{Ct value human }\beta \text{-actin-Ct value murine GAPDH}\right)}\right)}$. The relative expression was adjusted with proportion of human (versus murine) tissue calculated by 2^-(*Ct value human* β-*actin-Ct value murine GAPDH*)^. Remaining calculations were done by using the following formula 2^-(*Ct value relevant gene-Ct value murine GAPDH*)^.

### Statistical Analysis

Gaussian distribution was tested by D‘Agostino and Pearson omnibus normality test before significances were pointed out by using Mann-Whitney-U-test. Expression levels were compared between different samples as well as between different mERV. Statistical evaluation was performed using GraphPad PRISM software, version 5.02. The Pearson correlation coefficient was calculated for EcoMLV and EndoPP expression in PDX samples. *p*-Values lower than 0.05 were considered statistically significant.

## Results

Starting point of the present analysis were two CRC cases which could reproducibly trigger murine lymphoma development in immunodeficient NOD-SCID mice when engrafted as PDX. RNA-seq analysis identified several sequences with homology to viral sequences in both PDX (HROC32 and HROC39), one contig attributed to a papillomavirus, and other hits matching common NGS bacterial contaminants were also found, but will not be discussed further here (full data and sequences are available in **Supplementary Table S1**). No other viral sequences were detected.

**FIGURE 1:**
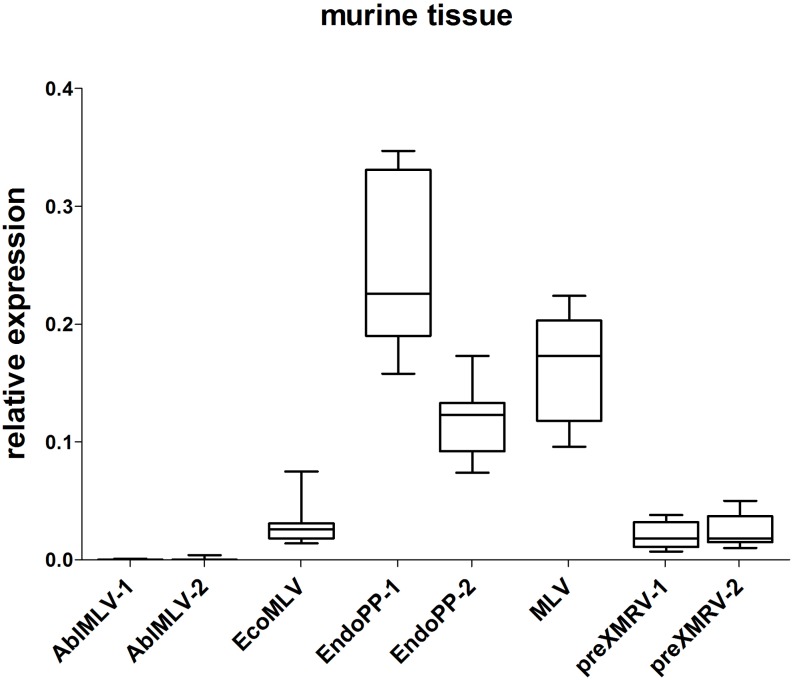
Relative expression of mERV in murine tissue. Expression levels were analyzed by SYBR Green based quantitative real-time (qRT-PCR) and the relative expression was calculated in relation to the murine housekeeping-gene GAPDH. The analyses of several healthy murine tissue (*n* = 11) from three different mouse strains (NMRI nu/nu, C57BL6, and NSG) shows clear expression differences between the different mERV: AblMLV was not expressed, EcoMLV and preXMRV showed moderate expression levels, whereas EndoPP was generally the highest expressed mERV with expression ranges from 0.074 to 0.347.

Those mERV detectable in both PDX cases were considered most relevant. These were: murine leukemia virus (MLV), AblMLV, ecotropic murine leukemia virus (EcoMLV), *Mus musculus* mobilized endogenous polytropic provirus (EndoPP), and pre xenotropic murine leukemia virus-related virus (preXMRV). For each candidate, two SYBR Green based qPCR primer systems were designed and tested in an initial screening experiment using cDNA from different normal murine tissues. Two primer systems delivered unspecific signals and were thus excluded from further analysis. **Figure 1** displays the relative expression of the investigated mERV in several healthy murine tissues (*n* = 11) from three different mouse strains. Clear expression differences between the different mERV became obvious. AblMLV was not expressed. EcoMLV and preXMRV showed moderate expression levels, whereas MLV and EndoPP were generally the highest expressed mERV. A generally even higher mERV activity was observed in murine tissues of C57 BL/6 origin, followed by NSG and then NMRI nu/nu mice (**Supplementary Figures S1A–C**). Yet, for all used mERV primer combinations signals were weaker than that of the murine housekeeping gene, GAPDH (0- to 0.4-fold relative expression).

**FIGURE 2:**
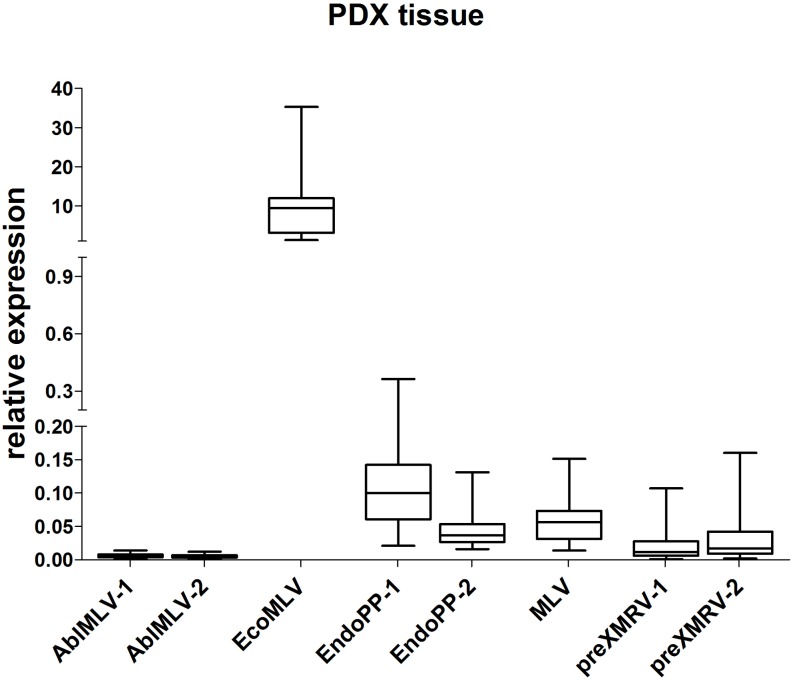
Relative expression of mERV in PDX from CRC. Expression levels were analyzed by SYBR Green based qRT-PCR. The analyses of *n* = 22 individual CRC samples shows clear relative expression differences between the different mERV with very weak signals from AblMLV, weak to intermediate from EndoPP, MLV, and preXMRV and highest from EcoMLV.

Next, the relative expression of these five mERV was analyzed in a small series of CRC PDX tissues (*n* = 22) (**Figure 2**). As expected, a variation in expression could be observed between the different mERV in the PDX tumor models on the one hand. Very weak signals came from AblMLV, weak to intermediate from EndoPP, MLV, and preXMRV and the single highest expressed mERV was EcoMLV. On the other hand, bigger differences in expression were observed for preXMRV and EcoMLV between individual PDX samples.

When directly comparing the mERV expression between murine control and PDX tissues, EcoMLV showed a significantly (*p* < 0.0001) higher expression in PDX than in murine control tissues, whereas EndoPP expression behaved just opposite (*p* < 0.0001).

According to definition, an ecotropic retrovirus like EcoMLV does not replicate in the original host but mainly in thereof established cell cultures. Thus, the observed high expression in the xenotransplanted tissue raises the question whether PDX may trigger EcoMLV expression, and dampen EndoPP expression. This was subsequently investigated in more detail.

**FIGURE 3:**
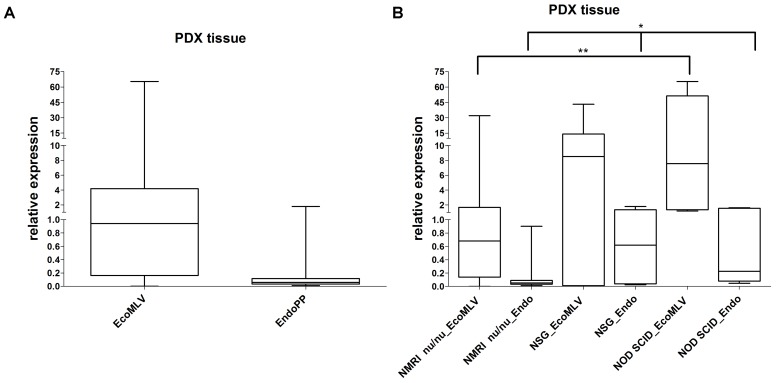
Relative expression of EcoMLV and EndoPP in PDX from CRC. **(A)** Expression levels were analyzed by qRT-PCR with probe systems specific for EcoMLV and EndoPP and relative expression was calculated by using the described formula. The analysis of *n* = 62 individual CRC PDX shows high expression of EcoMLV ranging from zero to 65.35. Expression of EndoPP was low, ranging from 0.01 to 1.79. Considerable differences in expression between individual PDX samples can be depicted. **(B)** Same expression data stratified according to the mouse strains, NMRI nu/nu (*n* = 50), NSG (*n* = 7), and NOD SCID (*n* = 5) used to generate the PDX. Significant differences in EcoMLV and EndoPP expression for NMRI nu/nu vs. NOD SCID (*p* = 0.0062 and *p* = 0.0140, respectively) (^∗∗^) and in EndoPP expression for NMRI nu/nu vs. NSG (*p* = 0.0196) are indicated (^∗^).

Probe systems specific for EcoMLV and EndoPP were designed and used to assess expression in the previously analyzed 22 PDX as well as in 40 additional CRC PDX (**Figure 3A**). Polymerase chain reaction (PCR) signals of EcoMLV were higher than the one of the housekeeping gene (4.80) but with a range between zero to over 60-fold. EndoPP PCR signals were much lower (0.209) with relative expression levels ranging from 0.01 to 1.79. Of note, expression levels of EcoMLV and EndoPP in the PDX were strongly positively correlated (*p* < 0.00001). Overall, the results of the probe systems confirmed the findings of the less-specific qPCR screening experiments (**Figure 2**).

We additionally compared expression in the different mouse strains used to generate PDX models (**Figure 3B**). Differences in expression were significant for EcoMLV and EndoPP in NMRI nu/nu vs. NOD SCID (*p* = 0.0062 and *p* = 0.0140, respectively) as well as for EndoPP in NMRI nu/nu vs. NSG (*p* = 0.0196). No significant difference was observed for EcoMLV expression in NSG vs. NMRI nu/nu (*p* = 0.0682) and vs. NOD SCID (*p* = 0.8763). EndoPP expression was also not significantly different between NSG and NOD SCID (*p* = 1.000). Thus, both mERV were expressed higher in NSG and NOD SCID compared to NMRI nu/nu mice.

Next, we analyzed, if expression of these two mERV is restricted to CRC-derived PDX by analyzing five PDX established from glioblastoma (**Supplementary Figure S2**). Here, the expression of EcoMLV and EndoPP showed no significant difference when compared to the ones of the CRC-derived PDX. Thus, we assume that at least expression of these two mERV cannot specifically be attributed to the PDX tissue origin.

**FIGURE 4:**
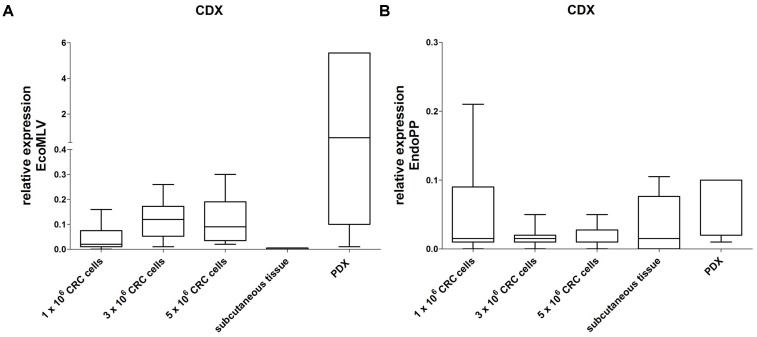
Relative expression of EcoMLV and EndoPP in CRC CDX. Expression levels were analyzed by qRT-PCR with probe systems specific for EcoMLV and EndoPP. CDX were generated by injecting 1, 3, and 5 × 10^6^ cells of *n* = 4 patient-derived CRC cell lines (without mERV expression) subcutaneously into NMRI nu/nu and NSG mice. Tissues were harvested and the relative mERV expression was determined as described. Subcutaneous control tissue and the corresponding PDX tissues are given as a reference. **(A)** Relative EcoMLV expression was generally higher in the CDX tissues than in the mouse control tissues, and tended to be lower than the expression in the reference PDX tissues from the same tumor cases. **(B)** Relative EndoPP expression was low with little variation between CDX, mouse control tissues and PDX.

In order to analyze if the human CRC cells are sufficient to influence mERV expression, we next generated xenografts from four patient-derived cell lines. Those were tested before to be free of mERV expression and were injected in different tumor cell numbers (1, 3, and 5 × 10^6^ cells) subcutaneously into NSG and NMRI nu/nu mice. The CDX tissues, obtained after several weeks of growth, were analyzed side by side with subcutaneous tissue from a CDX-free area of the same animals. In the CDX tissues, EcoMLV was generally expressed higher than in the CDX-free mouse tissues (**Figure 4A**). In direct comparison to the expression values obtained with the PDX tissues from the same tumor cases, the expression tended to be lower; this, however, was not statistically significant. EndoPP expression was very low with little variations between tumors and mouse tissues (**Figure 4B**).

**FIGURE 5:**
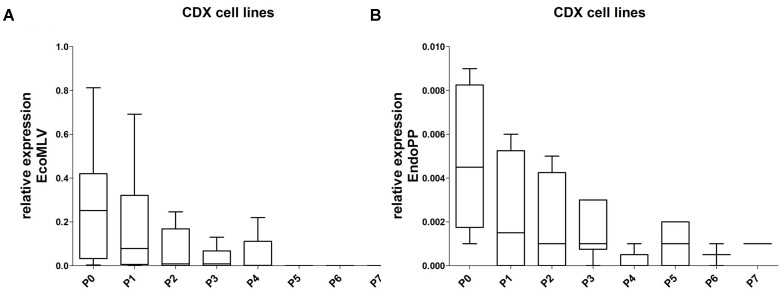
Relative expression of EcoMLV and EndoPP in re-cultivated cells from the CDX. Expression levels were analyzed by qRT-PCR with probe systems specific for EcoMLV and EndoPP. **(A)** The relative expression of EcoMLV in re-cultivated cells decreased gradually; down to zero at passage 5. **(B)** The relative expression of EndoPP in re-cultivated cells shows a similar gradual decrease; at passage 7, one sample gave a borderline signal.

The CDX tissues were additionally re-cultivated and expression of the two mERV was measured in these secondary cell cultures. Interestingly, EcoMLV as well as EndoPP were both initially expressed; but the levels decreased gradually with increasing passage numbers to or below the detection limit (**Figures 5A,B**). This observation might best be explained with mERV activity being attributable to the murine cells present in CDX (and PDX) tissues and they additionally suggest that no persistent infection of human tumor cells took place.

To address this question in more detail, we subsequently analyzed mERV expression in a panel of cell lines established in our lab from both primary CRC tissues (*n* = 17) and PDX tissues (*n* = 28) established from primary CRC cases, with *n* = 6 matching pairs. Generally, no expression of EcoMLV and EndoPP was observed in the CRC cell lines – of note, neither in the patient-derived nor in the PDX-derived cell lines.

A failure or bias of the detection systems used can be largely ruled out, since in several cell lines established from murine tumors, expression levels of EcoMLV and EndoPP corresponded to the levels observed in the PDX samples (**Supplementary Figure S3**).

Finally, we tested genomic DNA from these PDX-derived cell lines in an endpoint PCR using all available mERV primer systems. No amplicons were obtained from the probe system primers for EcoMLV and EndoPP (**Table 3**), but occasionally, bands were observed from several of the SYBR Green based primer systems used for the initial screening (**Table 1**). However, sequencing of these amplicons failed to detect any mERV-specific sequence. Thus, unspecific amplification must have taken place in some cases. In sum, we could not demonstrate integration into human tumor cells of any of the mERV analyzed after PDX-passaging in immunodeficient mice.

## Discussion

Patient-derived xenografts are considered the models best conserving the tumors’ original biological features including microarchitecture and micromilieu. Maybe more important, they also have the best clinical prediction value for individual treatment success (Guo et al., 2016). Thus, they are not only frequently used for basic research applications, but have recently re-emerged as ideal tool for preclinical drug development (Izumchenko et al., 2016).

However, it has repeatedly been observed that the xenografting procedure might result in mERV activation – at least in the PDX tissues (Todaro et al., 1973; Oakes et al., 2010; Delviks-Frankenberry et al., 2013; Naseer et al., 2015). Moreover, mERV activity has also been described in cell lines established from PDX (Zhang et al., 2011; Kirkegaard et al., 2016).

We here addressed the question to which extent mERV are activated and possibly transmitted to human CRC cells when passaged as PDX in immunodeficient mice. Technically, this approach was possible due to a large collection of low-passage, patient-derived tumor models including PDX and matching tumor- and PDX-derived permanent cell lines (Maletzki et al., 2015; Gock et al., 2016; Kuehn et al., 2016; Mullins et al., 2016).

Instead of analyzing pre-described mERV, a global NGS-screen for expressed mERV sequences was performed on two PDX observed to repeatedly induce leukemia of murine cell origin. This is likely to be a functional consequence of mERV (re)-activation. Triviai et al. (2014) investigated a similar hypothesis concerning induction of leukemia in a PDX model of primary myelofibrosis. They identified highly virulent polytropic MLV recombinants generated by incorporation of sequences from xenotropic ERV provirus as causative for this acute myeloid leukemia.

From all mERV sequences identified in our NGS screen, we selected five candidates found expressed in both PDX models analyzed; i.e., AblMLV, EcoMLV, EndoPP, MLV, and preXMRV. With the exception of AblMLV, all selected mERV were expressed in murine control tissues from three different mouse strains. In a large collection of PDX-tissues, all mERV were found expressed, even to a very low degree AblMLV. The expression levels largely varied in the PDX samples as well as in the murine tissues; whereby EcoMLV showed a significantly (*p* < 0.0001) higher expression in PDX than in murine control tissues. EndoPP expression behaved just opposite. According to these results, human tumors’ PDX seem to trigger EcoMLV but dampen EndoPP expression. This behavior of EcoMLV fits well to the host tropism of an ecotropic virus (Stoye and Coffin, 1987). However, we did not further analyze if this opposite behavior is functionally linked.

Moreover, compared to murine control tissues, EcoMLV was found to be higher expressed not only in PDX – independent of the origin of the transplanted human tumor tissue – but also in murine cell lines and in CDX.

An important and somewhat unexpected finding of the present study was that not only directly patient-tumor-derived but also PDX-derived CRC cell lines had no detectable mERV expression – even if the cell lines were established from PDX tissues with proven mERV (in particular EcoMLV) activity. This is in direct contrast to the findings of Zhang et al. (2011), who detected an infection with XMRV in 23% of PDX-derived cell lines and a 17% infection rate of human non-PDX-derived cell cultures; with the latter infections ascribed to horizontal spread *in vitro*.

Therefore, we also addressed the question, whether mERV expression is transient or more stable in cell cultures from freshly explanted human xenograft tissue with a series of CDX-experiments. In direct comparison to PDX, mERV expression is similar in CDX; but in CDX-derived secondary cultures it gradually disappears within a few passages. The best explanation for this finding is that mERV are expressed in the murine cells present in PDX tissues – and thus mERV expression vanishes with the decreasing contaminating murine cells. This result might partly explain the high contamination rate described by Zhang et al. (2011). But then again, CRC cells might be less susceptible for XMRV. So far, only one cell line isolate, i.e., the cell line RKO, has been found positive for XMRV in just one lab (Zhang et al., 2011).

Only high standards in tissue culture could prevent such contaminations. Though, even then, cross-contamination and horizontal transmission should not generally be neglected and further studies are needed to clarify which mERV (including mERV recombinants like XMRV) are incidental or frequent contaminants for tumor models of which tumor entities. Similar to identity testing, routine quality tests have to be developed and subsequently strictly applied to prevent data mis-interpretation when mERV-contaminated human tumor models are used.

Furthermore, our study pointed out that mERV integration into the genome of human tumor cells was not detectable and therefore seems to be unlikely or at least a rarer event than previously thought. Technical problems due to laboratory contaminations seem to be a serious issue in this context. Human DNA samples tested positive for XMRV/MLV sequences were found contaminated with murine DNA (Oakes et al., 2010). And even laboratory reagents containing trace amounts of murine nucleic acids have repeatedly been tested to cause false-positive XMRV detection results (Sato et al., 2010; Zheng et al., 2011).

Taken together, these findings imply that at least most mERV simply represent stowaways which are active within murine cells in PDX tissues and of early PDX-derived cell cultures. This is an important finding generally in favor of the utility of PDX and PDX-derived tumor models, since it attenuates the likelihood of a mERV bias permanently introduced into these models by the murine environment. The observed difference in mERV expression between PDX from different immunodeficient mouse strains additionally hints toward increasing mERV activity levels with the degree of immunodeficiency. Yet, none of these results can be generalized without testing of further immunodeficient mouse strains and PDX models of more tumor entities using similar global mERV screening strategies.

These facts highlight the importance of future research on this topic. Which mouse strain shall be selected for which experimental approach taking spontaneous and PDX-induced mERV activity and infections into account? What influences do specific mERV like EcoMLV and XMRV have on biological functions *in vivo*? It might also be speculated that mERV activations are somehow related to the success rate of PDX models and one might ask, if the higher level of mERV activation in strains with higher levels of immunodeficiency is worth the tradeoff for a better tumor take rate?

The influence of additional factors like bacterial and viral co-infections, feed, housing conditions, age, fitness, etc. on mERV activity might also be worth analyzing.

Finally, when considering the increasing number of co-culture protocols using two or more cell lines from different donors and laboratories (Miki et al., 2012), it will be wise to include mERV activity analysis under such conditions into standard quality control schemes.

The present study has some obvious constraints: only two PDX models have been used as starting point for the NGS-based global screening, leading to unambiguous (pro)virus identification by the short sequences obtained by NGS. Additionally, result of a qPCR-based analysis shall be validated on the protein level. Still, one can conclude that this comprehensive analysis of mERV activity in a large cohort of patient-derived CRC tumor models in low passages delivered in summary the following data: (i) PDX and murine tissues in general are likely to be contaminated by mERV – but variations between different immunodeficient mouse strains can readily be observed, (ii) mERV are expressed transiently and at low level in fresh PDX-derived cell cultures and gradually decrease to zero within a few passages, (iii) mERV integration into the human tumor cells’ genome is an unlikely or at least very rare event, (iv) “mouse free” cell cultures (i.e., cultures established from primary tumors) are free of any mERV activity.

## Ethics Statement

Specimen collection was conducted in accordance with the ethics guidelines for the use of human material, approved by the Ethics Committee of the University of Rostock (Reference numbers: II HV 43/2004, A45/2007, and A 2009/34) and with informed written consent from all patients prior to surgery.

Mouse breeding took place in the animal facilities (University of Rostock) under specified pathogen-free conditions. Trials were performed in accordance with the German legislation on protection of animals and the Guide for the Care and Use of Laboratory Animals (Institute of Laboratory Animal Resources, National Research Council; NIH Guide, Vol. 25, No. 28, 1996; Approval No.: LALLF M-V/TSD/7221.3-1.1-071/10 and LALLF M-V/TSD/7221.3-2-036/13).

## Author Contributions

SB conducted most of the molecular biology work, designed the probe systems, performed cell culture and the CDX experiments, and wrote the manuscript. EK critically reviewed the manuscript. PP performed the initial NGS screen and designed the primers used in the screening experiment. CM implanted the CRC cells into the immunodeficient mice. CSM was involved in the data analysis and language-edited the final version of the manuscript. ML designed the study and wrote the manuscript.

## Conflict of Interest Statement

The authors declare that the research was conducted in the absence of any commercial or financial relationships that could be construed as a potential conflict of interest.

## Supplementary Material

FIGURE S1 Relative expression of mERV in murine tissue stratified to mouse strains analyzed. Expression levels were analyzed by SYBR Green based qRT-PCR. The analysis of several healthy murine tissues shows the highest mERV activity in **(A)** C57BL6 followed by mERV expression in **(B)** NSG and then in **(C)** NMRI nu/nu mice.

FIGURE S2 Relative expression of EcoMLV and EndoPP in PDX tissue derived from glioblastoma (*n* = 5). Expression levels were analyzed by SYBR Green based qRT-PCR. The relative expression of EcoMLV ranged from 3.31 to 527.58 and of EndoPP from 0.09 to 0.84.

FIGURE S3 Relative expression of EcoMLV and EndoPP in murine cell lines (*n* = 5). Expression levels were analyzed by SYBR Green based qRT-PCR. The relative expression of EcoMLV ranged from16.29 to 94.58 and of EndoPP from 0.01 to 0.21.

TABLE S1 MERV sequences identified by NGS.
