# Supplementary figures and images for: Murine Endogenous Retroviruses Are Detectable in Patient-Derived Xenografts but Not in Patient-Individual Cell Lines of Human Colorectal Cancer
Source: Front Microbiol. 2018 Apr 24;9:789. doi: 10.3389/fmicb.2018.00789 (PMC5932414; doi:10.3389/fmicb.2018.00789)

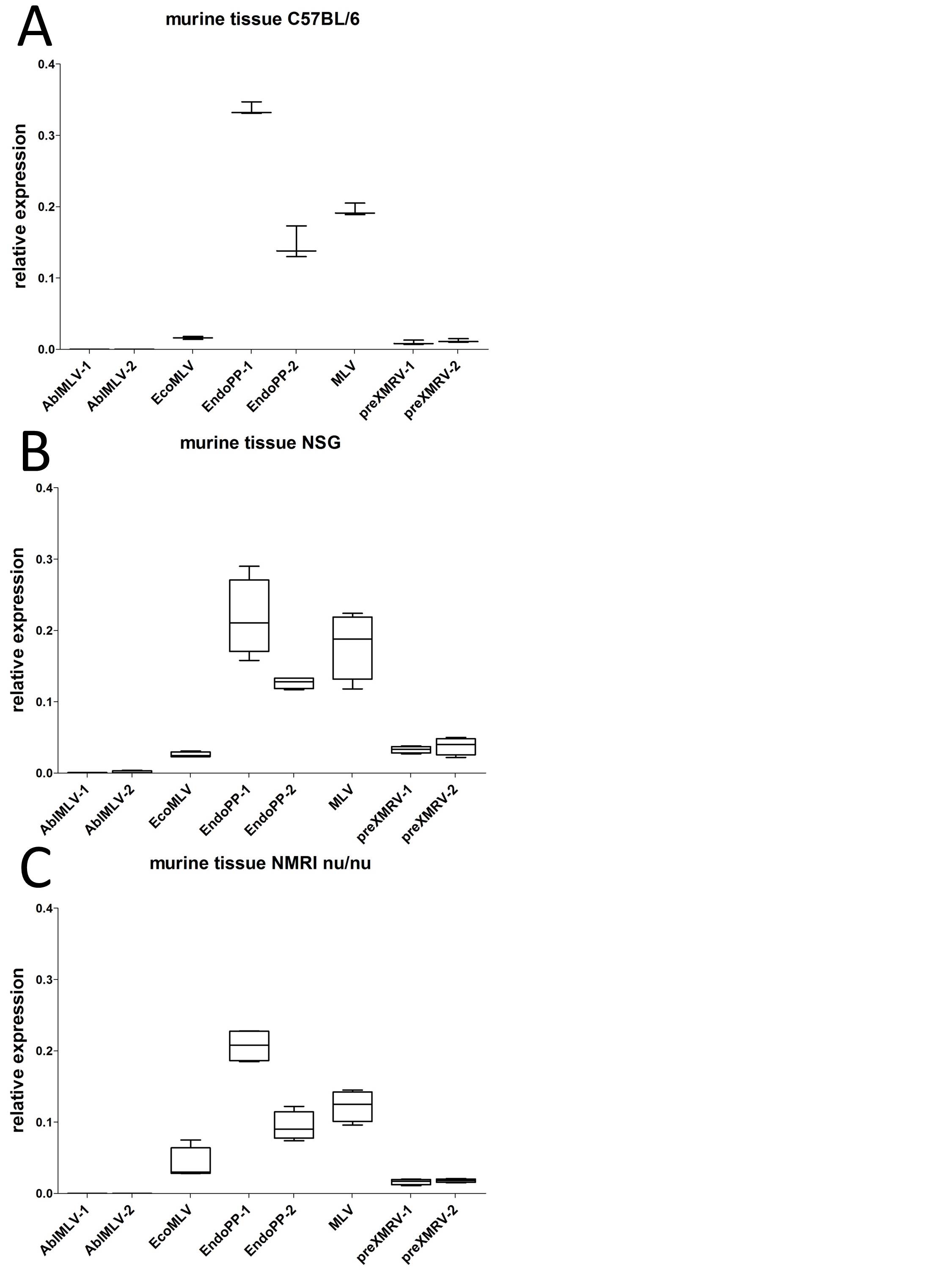

Supplement: FIGURE S1 — Relative expression of mERV in murine tissue stratified to mouse strains analyzed. Expression levels were analyzed by SYBR Green based qRT-PCR. The analysis of several healthy murine tissues shows the highest mERV activity in (A) C57BL6 followed by mERV expression in (B) NSG and then in (C) NMRI nu/nu mice. [file Image_1.JPEG]

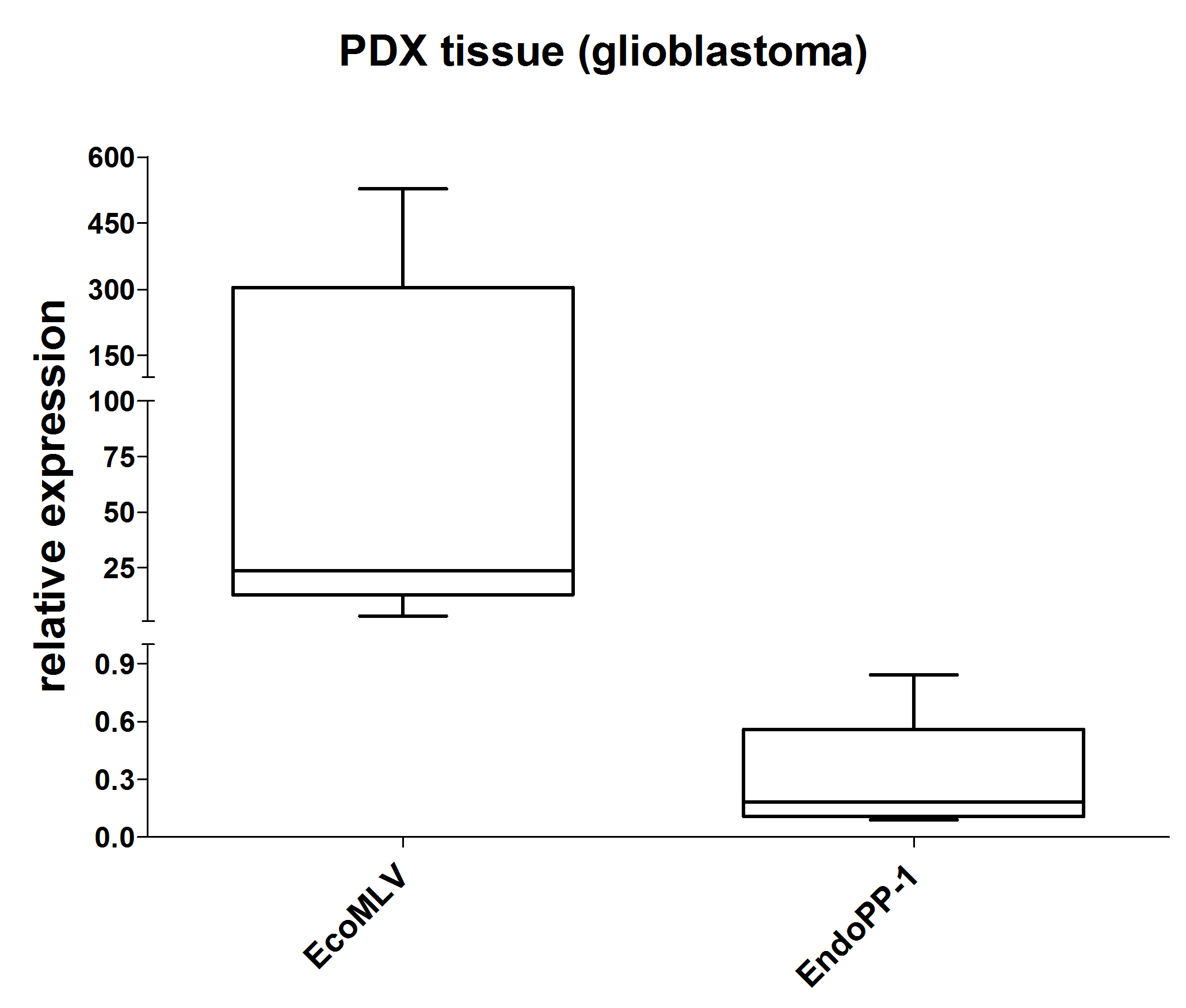

Supplement: FIGURE S2 — Relative expression of EcoMLV and EndoPP in PDX tissue derived from glioblastoma (n = 5). Expression levels were analyzed by SYBR Green based qRT-PCR. The relative expression of EcoMLV ranged from 3.31 to 527.58 and of EndoPP from 0.09 to 0.84. [file Image_2.JPEG]

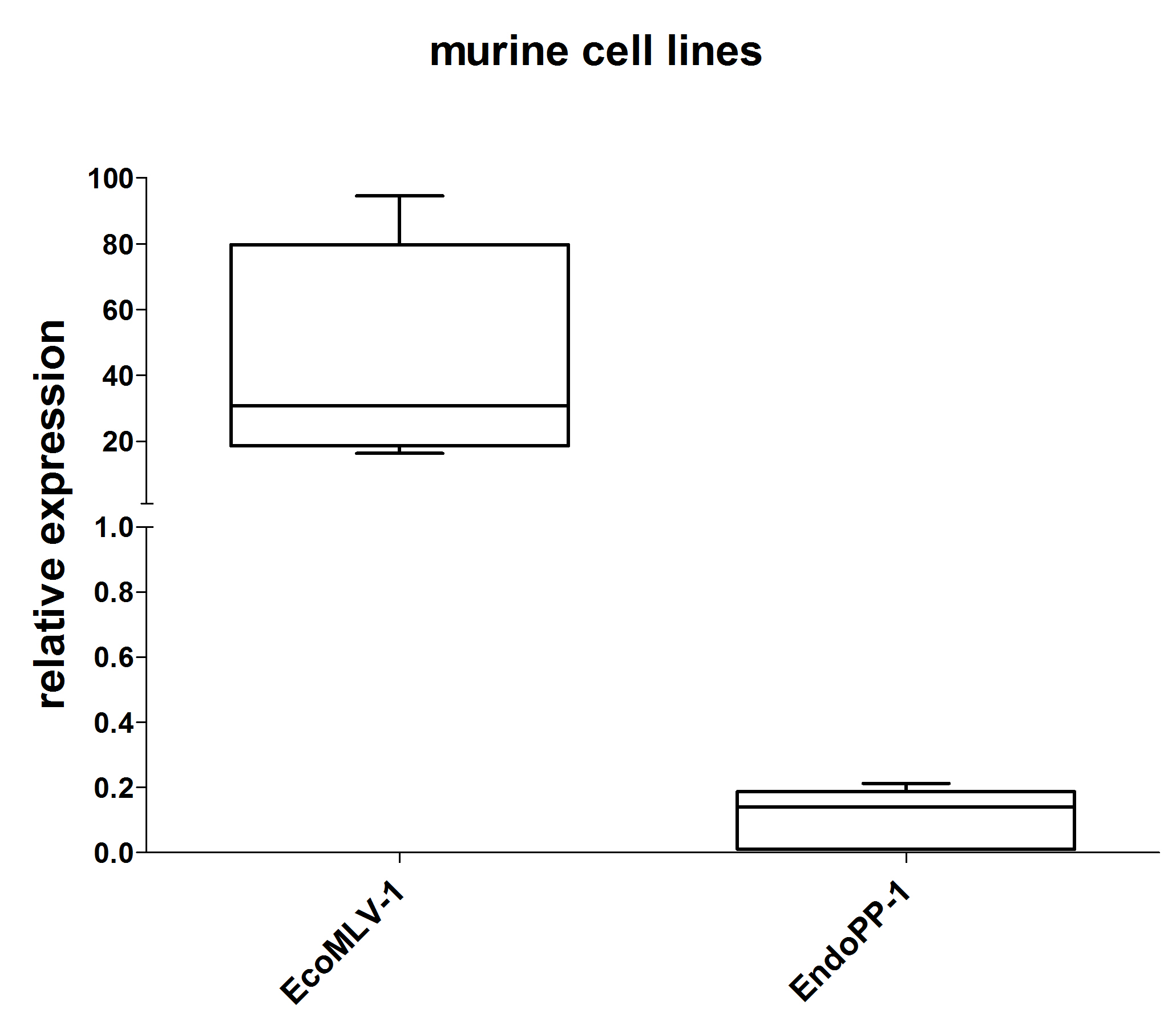

Supplement: FIGURE S3 — Relative expression of EcoMLV and EndoPP in murine cell lines (n = 5). Expression levels were analyzed by SYBR Green based qRT-PCR. The relative expression of EcoMLV ranged from16.29 to 94.58 and of EndoPP from 0.01 to 0.21. [file Image_3.JPEG]
